# Reconstructing vapor pressure deficit from leaf wax lipid molecular distributions

**DOI:** 10.1038/s41598-018-21959-w

**Published:** 2018-03-02

**Authors:** Yvette L. Eley, Michael T. Hren

**Affiliations:** 1 Center for Integrative Geosciences, University of Connecticut, Storrs, Mansfield, CT 06269 USA; 2 Department of Chemistry, University of Connecticut, Storrs, Mansfield, CT 06269 USA; 3 Present Address: School of Geography, Earth and Environmental Sciences, University of Birmingham, Edgbaston, Birmingham B15 2TT UK

## Abstract

Estimates of atmospheric moisture are critical for understanding the links and feedbacks between atmospheric CO_2_ and global climate. At present, there are few quantitative moisture proxies that are applicable to deep time. We present a new proxy for atmospheric moisture derived from modern climate and leaf biomarker data from North and Central America. Plants have a direct genetic pathway to regulate the production of lipids in response to osmotic stress, which is manifested in a change in the distribution of simple aliphatic lipids such as *n-*alkanes. The Average Chain Length (ACL) of these lipids is therefore statistically related to mean annual vapor pressure deficit (VPD_av_), enabling quantitative reconstruction of VPD from sedimentary *n-*alkanes. We apply this transfer function to the Armantes section of the Calatayud-Daroca Basin in Central Spain, that spans the Middle Miocene Climatic Optimum (MMCO) and the Middle Miocene Climate Transition (MMCT). Reconstructed VPD_av_ rises from 0.13 to 0.92 kPa between 16.5 and 12.4 Ma, indicating a substantial drying through the MMCT. These data are consistent with fossil assemblages and mammalian stable isotope data, highlighting the utility of this new organic molecular tool for quantifying hydrologic variability over geologic timescales.

## Introduction

The distribution of ecosystems across the globe is strongly regulated by the availability of water^1^. In the face of predicted future climate shifts, reconstructions of past climate and hydrology provide critical opportunities to evaluate the relationship between atmospheric CO_2_ and hydrological regimes during periods when Earth was significantly warmer than today^2^. At present, however, reconstructions of past water availability suffer from large uncertainties, especially in regions that are prone to large fluctuations in climate and hydrology in response to variations in *p*CO_2_ (e.g. Western North America).

A number of semi-quantitative geochemical methods have been used to infer paleoaridity. These include: (i) stable isotopes of fossil bones/teeth^3,4^, speleothems^5^, and soil carbonates^6,7^; (ii) the presence and/or provenance of dust in ice cores^8^; (iii) the carbon isotope composition of plants^9,10^; and (iv) the geochemical composition of paleosols^11,12^. While these methods have advanced our understanding of paleohydrological processes, they possess a number of significant mechanistic, temporal or spatial limitations. For example, dust in ice cores is strongly influenced by changes in wind strength and direction, in addition to aridity^13^. Ice cores also only record the last few million years of glacial conditions, placing important temporal constraints on paleoclimatic reconstructions. Conflict between paleosol and fossil bone carbonate δ^18^O records^4,12^ is likely due to the fact that the isotopic composition of ungulate tooth enamel can record the influence of a complex mixture of factors such as temperature, water availability and atmospheric circulation patterns^14^, as well as the ability of animals to migrate significant distances to continue residing in their ideal habitat^15^. Furthermore, soil carbonates only form in a discrete range of hydrologic conditions. A shift from a dry environment (characterized by soil carbonate formation) to wetter conditions is often marked by a reduction or complete absence of soil carbonate nodules.

**Figure 1:**
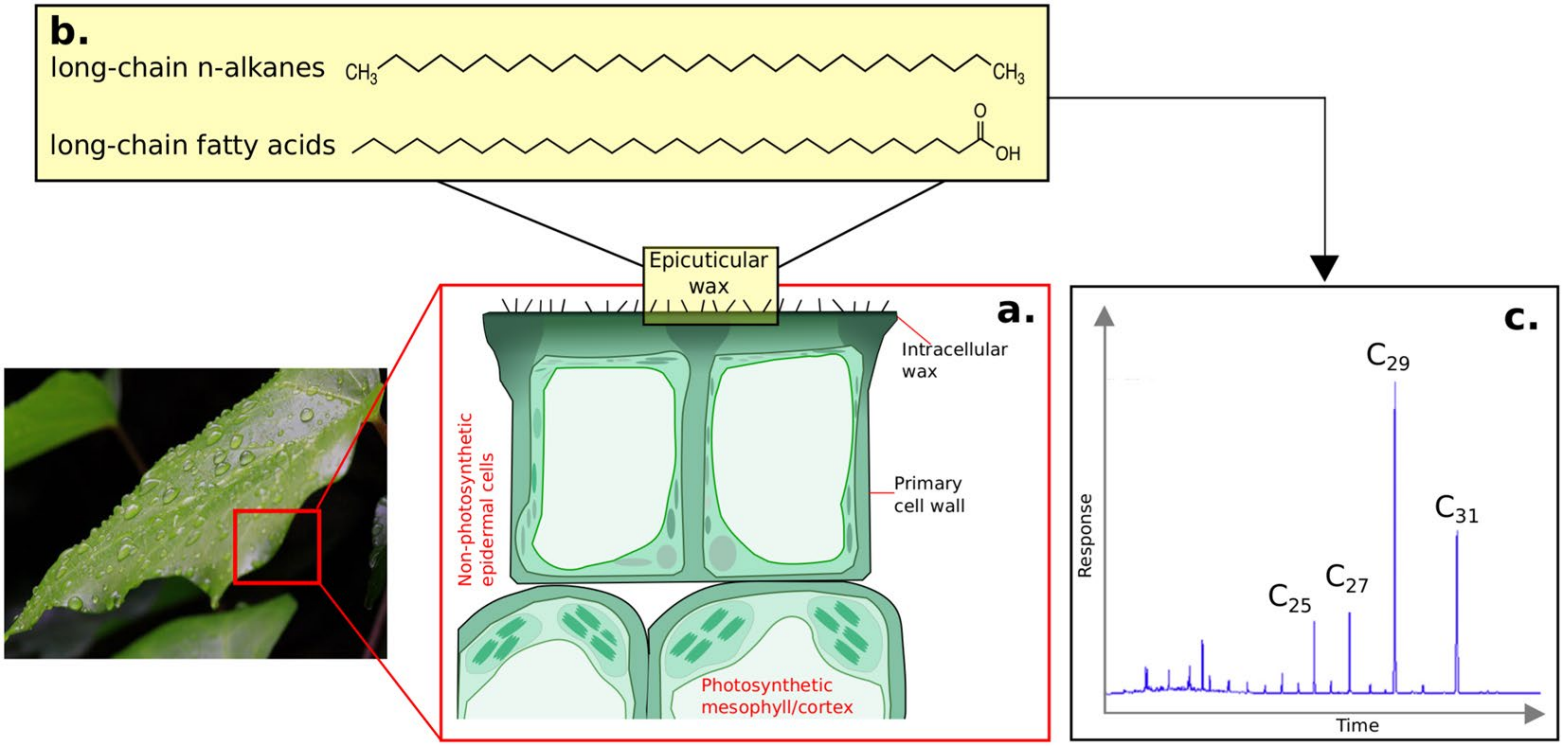
Schematic showing the morphology and chemistry of leaf epicuticular waxes: (**a**) transverse view of the epidermal cells showing intracellular and epicuticular wax, alongside the cell wall and other photosynthetic and non-photosynthetic cell arrangements (figure adapted from^68^ with the permission of Elsevier); (**b**) long chain *n-*alkanes and fatty acids, ubiquitous compounds in leaf waxes from terrestrial higher plants; and (**c**) typical chromatogram of leaf wax *n-*alkanes from terrestrial higher plants showing the distribution of long-chain homologues. Leaf photograph shown is reproduced under CC0 1.0.

In recent decades, there has been a dramatic upsurge in the use of molecules present in the epicuticular waxes coating leaves of terrestrial and aquatic higher plants (Fig. 1) as proxies for paleoclimatic and paleoenvironmental reconstructions^16^. The are many advantages to this biomarker-based approach. *n-*Alkyl lipids from higher plants are found in both terrestrial and marine sedimentary systems^10,17–20^, and are relatively resistant to shallow burial diagenesis^21–23^, enabling paleoenvironmental reconstruction over hundreds of millions of years of Earth history^24,25^. A number of methods have been used to infer paleoclimatic information from leaf wax biomarkers. The δ^13^C compositions of *n-*alkanes in sedimentary archives are commonly used to identify the proportion of C_3_ and C_4_ plants, and hence extrapolate changes in temperature and aridity^26,27^. Relative humidity and the availability of moisture are also thought to be important determinants of the relationship between the hydrogen isotope composition of precipitation and the δD signal recorded by leaf waxes^28^. The extent that leaf wax *n-*alkane δD values record this aridity/transpiration signal, however, remains controversial. Some studies of modern vegetation suggest that transpiration influences *n-*alkane δD values^29^, yet grasses grown under controlled greenhouse conditions show no *n-*alkane δD shifts in response to changes in relative humidity^30^. In addition, biochemical processes are shown to drive interspecies variation in *n*-alkane δD among modern plants^31,32^, suggesting that mechanisms controlling leaf wax lipid hydrogen isotope compositions are not yet fully constrained.

The molecular distribution of leaf wax *n*-alkanes, commonly described by indices such as the Average Chain Length (ACL), is shown to vary along a latitudinal gradient, a phenomenon previously interpreted to be driven by changes in temperature^33–35^. However, correlation does not necessarily imply causation. ACL is also known to be related to changes in the availability of moisture, with longer chain lengths prevalent in more arid environments^34,36–38^. The trend for plants to synthesize longer alkane homologues under conditions with lower humidity has even been documented during glacial periods, where aridity is synchronous with colder climates^39^.

There are several ways to express the moisture state of the atmosphere. Relative humidity (rH), for example, describes the ratio of the actual vapor pressure of the air, (*e*_*a*_) and the saturation vapor pressure of air at a given temperature, *e*_*s*_(*T*_*a*_), and is reported as a percentage as shown in Equation 1 ^40^:

1 $${RH}=100\times \,\frac{{e}_{a}}{{e}_{s}({T}_{a})}$$

Relative humidity is therefore not an actual measurement of the quantity of water vapor in the atmosphere, but a simple ratio of two known values^40,41^. The actual water holding capacity of the atmosphere, however, doubles exponentially for every ~11 °C of temperature increase, meaning the same relative humidity over a range of temperatures can reflect very different atmospheric moisture contents^41^. In contrast, vapor pressure deficit (VPD) reports an absolute measure of the amount of atmospheric moisture relative to *e*_*s*_(*T*_*a*_) which is a function of temperature. VPD is defined in Equation 2 ^40^:

2 $${VPD}={e}_{s}({T}_{a})-{e}_{a}$$

VPD reflects the atmospheric moisture deficit, which controls the extent to which the atmosphere can extract moisture from land surfaces (and by extrapolation the evaporative demand on plants). If all other factors (e.g. windspeed, water availability) are constant, for a given VPD the rate of evaporation is constant regardless of air temperature^40^. A VPD of 2 kPa will therefore have the same impact on the rate of evaporation from plant leaves regardless of MAT - indeed, this is why VPD is argued to be a better determinant of plant water stress than rH^40,41^.

Just as plants respond to variation in the intra- vs. extracellular pressure in CO_2,_ the gradient between atmospheric and intracellular or leaf surface concentration of specific molecules (including water), influences the flux of volatile and organic compounds from plant leaves^42,43^. In addition, gene regulation within plants is shown to be sensitive to specific biologic triggers related to water availability, not temperature^44^. Here, we show that ACL records changes in VPD at the ecosystem scale, based on the statistically significant relationship between ACL and mean annual VPD (VPD_av_) across a range of biomes in North and Central America. We then apply this transfer function to an organic molecular record from Miocene sediments in Central Spain that span the Middle Miocene Climatic Optimum (MMCO) and the Middle Miocene Climate Transition (MMCT). This new paleohydrological tool enables reconstruction of VPD during this global climate transition.

## Sampling and Statistical Analysis

### Modern calibration studies

**Figure 2:**
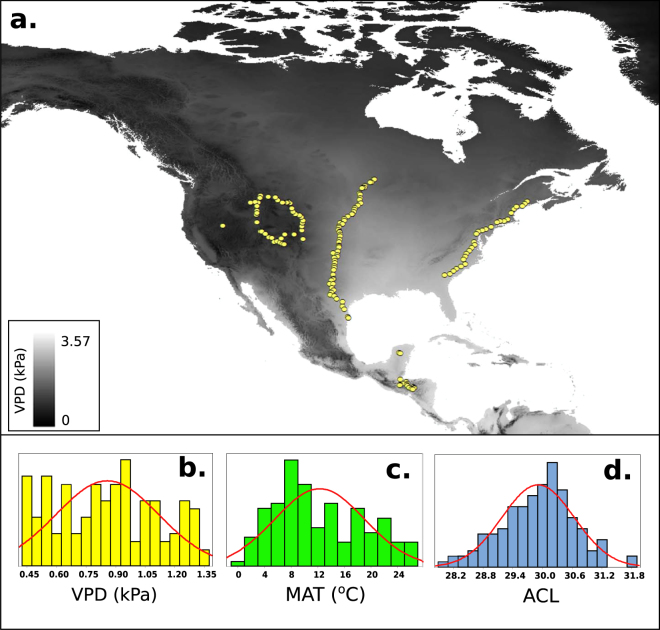
Distribution of surface soil locations in North and Central America used to derive the relationship between ACL and VPD. (**a**) Sample locations plotted against 30 year mean vapor pressures for July (data is a spatial interpolation of gridded WorldClim 2 data^46^, and the map was produced using ArcMap^69^. (**b**–**d**) Distribution of VPD, MAT and ACL values at our sites.

We compiled a comprehensive dataset of 149 new and previously published soil *n*-alkane profiles from North and Central America (Fig. 2; Table S1). Soils span a range of Koppen climates, from Koppen number 12 to 43^45^. We obtained climate variables from the ‘PRISM’ database (PRISM Climate Group, 2010), and the WorldClim 2 database^46^ (Supplementary Information). Both of these databases have a spatial resolution of ~800 m to 1 km grid squares, and provide 30 year normals of climate parameters such as monthly average temperature, precipitation and vapor pressure deficit. Sample sites span a range of ecosystem types with MAT from −0.2 to 26.6 °C, while ACL values from our selected soils range from 28 to 33, spanning the range of values commonly reported in sedimentary lipid biomarker studies^34,47^. Importantly, we focus on the ACL recorded in soils and sediments, which integrate vegetation inputs totalling hundreds or thousands of years, providing a combined ecosystem-scale signal rather than that of any individual plant species. Mean annual VPD values for all modern sites range from 0.4 kPa to 1.3 kPa. All statistical analyses describing the relationships between our data are carried out using Minitab v. 17.

To establish the relationship between sedimentary leaf wax *n*-alkane Average Chain Length (ACL) values and annual average vapor pressure deficit (VPD_av_) we combined previously published soil *n*-alkane distribution profiles from North and Central America^33,35,48^, with new measurements of soils and sediments from western North America (SI Table 1). Where possible, we recalculated this index from peak area determinations provided in previous publications using Equation 3 (where *C*_*x*_ refers to the peak area of the individual alkane), to ensure consistency in the alkane chain lengths used:

3 $${ACL}=\frac{{C}_{25}(25)+{C}_{27}(27)+{C}_{29}(29)+{C}_{31}(31)+{C}_{33}(33)+{C}_{35}(35)}{{C}_{25}+{C}_{27}+{C}_{29}+{C}_{31}+{C}_{33}+{C}_{35}}$$

We note the existence of a broad positive relationship between VPD and MAT in our sampling locations (Fig. S2). However, the presence of a genetic signalling pathway upregulating the production of leaf wax precursors in response to water deficit^44^, and the absence of a similar molecular response to increasing temperatures, leads us to conclude that it is VPD, rather than MAT, that is the dominant control on our ACL values.

## Results and Discussion

### Relationship between ACL and VPD in Modern Soils

**Figure 3:**
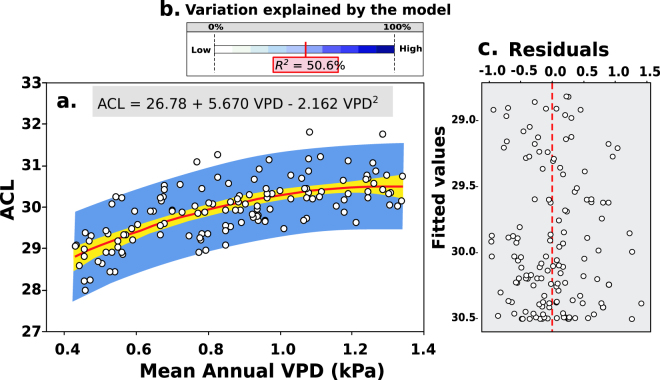
The relationship between Average Chain Length (ACL) and Vapor Pressure Deficit (VPD) (**a**) Statistical relationship between Average Chain Length (ACL) and mean annual vapor pressure deficit (VPD). (**b**) Strength of the relationship between ACL and VPD. (**c**) Residuals plot.

We used ACL and climate information from modern soils that span North and Central America to quantify the relationship between chain length distribution and VPD (Supplementary Information Table S2). We performed regression analysis using VPD as the predictor variable and ACL as the response variable, and identified a statistically significant (*p* < 0.05) positive relationship (Fig. 3) between ACL and VPD, described by Equation 4:

4 $${\rm{ACL}}=26.78+5.670\,{\rm{VPD}}-2.162\,{{\rm{VPD}}}^{2}$$

Our data show that half of the variation in ACL can be explained by variation in VPD. The residuals plot (Fig. 3b) displays homoscedacity, indicating that errors associated with the model predictions are stochastic. The standard error associated with VPD values calculated using our regression model is ±0.1 kPa. This error was subsequently assumed for all VPD reconstructions from the Miocene sediments.

### Proposed mechanism driving relationship between ACL and VPD

There are two primary mechanisms that could account for the observed relationship between ACL and VPD. The ‘plant production’ mechanism requires that plants actively shift towards preferential production of the longer-chain *n*-alkane homologues via the acetogenic lipid pathway (thus increasing ACL) in response to external environmental or climatic drivers^49^. Although the overall composition of leaf wax is genetically regulated and highly responsive to developmental and environmental factors^50^, previous studies show conflicting results regarding whether *n*-alkane distribution patterns vary dynamically and systematically in response to environmental change. A number of authors suggest that ACL is primarily linked to temperature^34,51^, however there is no specific biologic mechanism linking temperature to the chain length of compounds that are produced. But water deficit and osmotic stress stimulate transcription of the *CER6* gene, which is involved in the elongation of fatty acid acyl-CoAs longer than C_22_^44,50^. This means that, unlike for temperature, there is a specific biologic signalling system that relates leaf wax biosynthetic processes to water deficit. Further, examination of our temperature and VPD data for all sampling sites show that these are linearly related (Supplementary Information Fig. S2), thus apparent links between ACL and MAT are most likely dominantly driven by the moisture deficit effects, rather than temperature alone.

The relationship between ACL and VPD does not require the invocation of a plant physiological or biochemical mechanism, however, as it can be explained entirely through physical processes. *n*-Alkanes are known to be released from plants alongside other volatile compounds (e.g., terpenes, isoprene) and water^52,53^. When VPD increases, evaporation of these volatiles from leaves rises, as plants regulate stomatal and cuticular transpiration in response to changes in the moisture availability of the atmosphere^43^. Longer chain length homologues have higher molecular masses than shorter-chain *n*-alkanes (e.g., nonacosane = 408.799 g/mol, pentacosane = 352.691 g/mol and tricosane = 324.637 g/mol), and evaporate more slowly due to stronger inter-molecule bonds^54^. As a result, shorter homologues are likely to be preferentially lost when VPD increases, potentially contributing to the observed positive relationship between ACL and VPD.

Sedimentary *n-*alkanes record the combined effect of VPD on many individual plants over hundreds and/or thousands of years. Thus, while modern studies of individual plant *n-*alkane responses to environmental perturbations may be highly specific and display a wide scatter, sedimentary alkanes record long-term ecosystem-scale changes due to climatic and hydrologic shifts. Further, should plants that preferentially produce longer-chain alkanes (as a protection against increasing aridity) proliferate as an ecosystem becomes more moisture depleted, soils will incorporate this vegetation shift signal over long formation timescales. Thus, as we specifically focus on soils here in our modern calibration, we effectively encompass this potential ‘ecosystem-change’ driver of the observed relationship between ACL and VPR in our transfer function.

### Paleoaridity changes during the MMCO and MMCT in Central Spain

The Miocene was a time of rapid climate shifts. The Middle Miocene Climatic Optimum (MMCO, 15–17 Ma) was the warmest period of the Neogene with temperatures some 3–8 °C warmer than present^55^. In contrast, the subsequent Middle Miocene Climate Transition (MMCT, ~15–13.7 Ma) saw the widespread expansion of the East Antarctic Ice Sheet, and a shift towards much cooler conditions^56^. On a global scale, changes in atmospheric *p*CO_2_ during the Miocene are likely to have played a dominant role in climate dynamics^57^. At a regional level, however, spatial heterogeneity in the nature and magnitude of environmental change suggests that more localised mechanisms such as tectonic uplift, changes in regional geology, shifts in temperature gradients or variation in freshwater inputs also influence terrestrial temperature and hydrology across Europe^58^. The production of new high-resolution sedimentary sequences spanning the MMCO and the MMCT, and the development of new organic molecular tools for paleoclimate reconstruction, are critical steps in the evolution of our understanding of terrestrial environmental change during the mid and late Miocene.

The Armantes section is located in the Calatayud-Daroca basin of central Spain and contains expanded sections of fluvial and paleosol sequences spanning the MMCO^59^ and the cooling associated with the MMCT^57,60,61^. Sedimentation is continuous, with no observed hiatuses between 17 Ma and 12 Ma^62^. The section is up to 280 m thick, consisting of alternating red clay/silts and pink/white indurated silty limestones for much of its expanse^59^. The Calatayud-Daroca basin contains many fossil bearing sediments, making it a key location for understanding the response of ecosystems in Southwest Europe to Miocene climate shifts^58^. The age model for this section is based on well-established magnetostratigraphy^59,62^. A number of paleobiological studies establish that many parts of Spain experienced widespread cooling and drying during this time interval^60,63^, making it an ideal sequence to evaluate our new leaf wax biomarker-based paleoaridity proxy and expand existing paleohydrological reconstructions of the MMCO and MMCT in central Spain.

**Figure 4:**
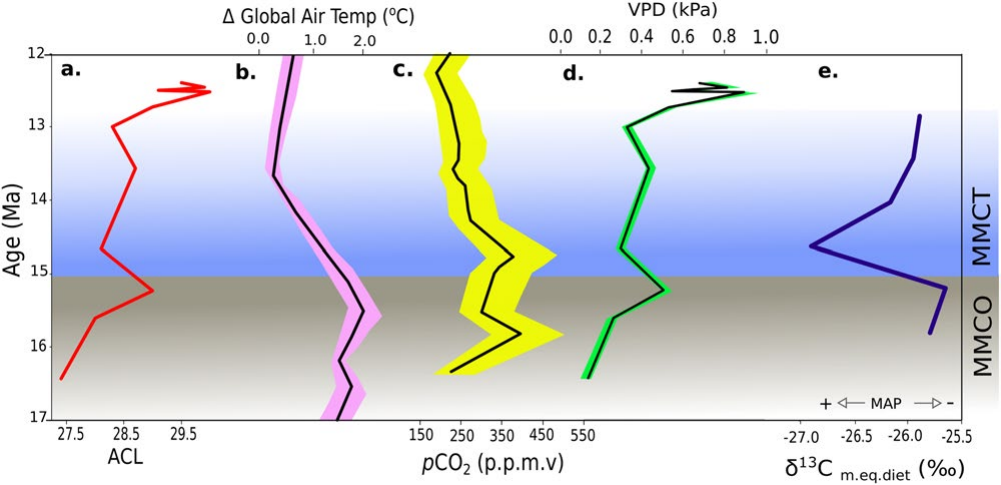
Average chain length (ACL) derived reconstruction of moisture availability during the MMCO and MMCT in Spain compared with other proxies for paleoaridity: (**a**) Leaf wax ACL values (this study). (**b**) Changes in global air temperature^61^. (**c**) Atmospheric *p*CO_2_^57^. (**d**) Reconstructed VPD_av_ (this study). (**e**) δ^13^C of modern equivalent diet used to reconstruct mean annual precipitation ‘MAP’^60^.

Sediments from the Armantes section record a positive shift of ~3 ACL units between 16.4 and 12.4 Ma. These sediments span > 200 m of stratigraphic section, and are unlikely to have experienced differential diagenesis between the top and bottom of the section following burial. Equally, despite sediments containing both paleosol and floodplain deposits, there is no indication that the observed changes in ACL are due to an increased contribution from wetland/aquatic plants. Indeed, aquatic plants frequently have distinctive *n*-alkane distribution patterns with carbon chain maxima at C_23_ or C_25_^64^, while all of the Armantes samples analysed here (with the exception of the sample dating from 16.4 Ma) have carbon chain maxima of C_29_ or C_31_. Rearranging Eq. 4 as shown in Eq. 5, we calculate reconstructed VPD values varying from 0.13 to 0.92 kPa during this interval, with higher values occurring at ~15.2 Ma during the MMCO, and again between 13.5 and 12.5 Ma, commencing during the MMCT and continuing into the late Miocene (Fig. 4).

5 $${\rm{VPD}}=1.3125-\sqrt{14.1208-0.4629\,{\rm{ACL}}}$$

Comparison of these data with previously existing paleoclimate reconstructions show good agreement, with relative decreases and increases in VPD_av_ occurring broadly in synch with decreases in global air temperature^61^, atmospheric *p*CO_2_^57^, and previous reconstructions of aridity in the region based on fossil tooth assemblages and stable isotope data^60,65^. We note, however, that the largest shift in reconstructed VPD occurs after 13 Ma, rather than during the MMCT interval (Fig. 4c). Reconstructions of mean annual precipitation in Europe suggest that rainfall declined throughout the late middle to late Miocene, and hence our increase in VPD (suggestive of a reduction in available moisture), is likely to reflect the broader aridification of Central and Southwest Europe previously identified between 13 and 11 Ma^58^. Meta-analysis of paleoprecipitation data from Europe suggests that such changes in rainfall are not directly correlated with global temperatures, but rather indicative of the influence of processes such as changes in ice-volume, shifts in hemispherical temperature gradients, and regional geography and topography^66^.

Analysis of modern sediment *n*-alkane profiles show that the distribution of homologues is strongly controlled by the moisture deficit of the environment. There are both biological and physical bases for this response, but most critically organic biomarkers in the sedimentary system record these processes allowing for quantitative reconstruction of paleo-VPD. This new organic molecular tool for paleohydrologic investigations provides new constraints on VPD in Central Spain during a critical climate transition, highlighting the applicability of this technique over geologic timescales.

### Analytical methods

#### Lipid extraction and quantification

We collected materials from fresh exposed paleosol and floodplain sediments from the Armantes section of the Calatayud-Daroca Basin in Central Spain deposited from the peak of MMCO warming (~16.5 Ma) to the MMCT transition period (~12.4 Ma). Modern surface soils collected as part of this study were collected from the top ~10 cm, with visible plant and root material removed. Soils and sediments were freeze dried as soon as possible after collection to minimise the potential for microbial alteration of sedimentary lipids^67^.

All surface soils and Miocene sediments analysed as part of this study were solvent extracted to obtain the aliphatic fraction containing *n-*alkanes. Approximately 150 g of dried sediment was extracted with soxhlet apparatus, using a 2:1 (v/v) mixture of dichloromethane and methanol. Extracts were concentrated under a stream of N_2_ gas, and then separated into compound fractions by silica gel chromatography using ashed Pasteur pipettes packed with activated silica gel (70–230 mesh). Aliphatic, aromatic and polar solutions were eluted by the sequential application of 2 ml hexane, 4 ml dichloromethane, and 4 ml of methanol. The hexane fraction was further purified using urea adduction. Adducted normal alkanes were extracted into hexane, concentrated under N_2_, and analyzed for molecular distributions on a Thermo-Scientific Trace GC Ultra equipped with a split-splitless injector and a flame ionisation detector, using a DB-5 column (60 m × 0.25 mm i.d., 0.25 µm) with helium as a carrier gas. Alkane peaks were identified by comparing retention times to those of a laboratory standard, and the Average Chain Length (ACL) was calculated using Eq. 3, and results are shown in Table S2.

### Data availability

All data generated or analysed during this study are included in this published article (and its Supplementary Information files).

## Electronic supplementary material

Supplementary Information
