# Supplementary material for: Reconstructing vapor pressure deficit from leaf wax lipid molecular distributions
Source: Sci Rep. 2018 Mar 2;8:3967. doi: 10.1038/s41598-018-21959-w (PMC5834636; doi:10.1038/s41598-018-21959-w)
Supplement: Supplementary file 1 — Supplementary Information [file 41598_2018_21959_MOESM1_ESM.pdf]

Supplementary Information

Reconstructing vapor pressure deficit from leaf wax lipid molecular distributions

Yvette Eley, Michael Hren

We collected a comprehensive dataset of 149 new and previously published soil *n*-alkane profiles from North and Central America, as detailed in Table S1. We obtained mean annual VPD data for each of our North American sample collection sites from the 'PRISM' database (PRISM Climate Group, 2010). For the Central American sites, we calculated this parameter from climate data provided by 'WorldClim 2' (Fick et al., 2017). We noted a systematic, consistent offset between PRISM and WorldClim 2 derived VPD. In order to standardise data to PRISM, we created a transfer function (Fig. S1) to standardize WorldClim 2 derived VPD values to the PRISM VPD scale prior to statistical analysis (Equation S1):

$$\text{PRISM VPD} = 0.3642 + 1.109\ \text{WorldClim 2 VPD} \qquad \text{Eq. S1}$$

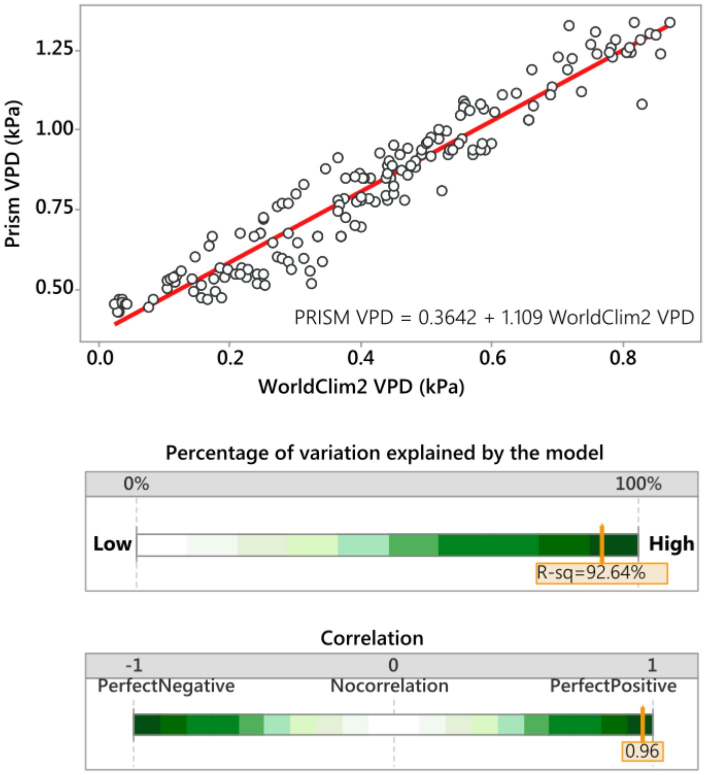

Figure S1: Regression model to standardize WorldClim 2 VPD and PRISM VPD values

Table S1: Modern compilation of soil data used to define the relationship between ACL and VPD

Data Source	Latitude	Longitude	ACL	VPD_{av} (kPa)	MAT (°C)
Bush and McInerney, 2015	48.60	-93.23	29.0	0.43	3.1
Bush and McInerney, 2015	48.58	-93.38	29.1	0.43	3.0
Bush and McInerney, 2015	46.14	-95.58	28.6	0.44	5.0
Bush and McInerney, 2015	48.08	-93.96	28.2	0.45	3.3
Bush and McInerney, 2015	47.22	-94.86	28.8	0.45	3.9
Bush and McInerney, 2015	47.20	-95.26	29.0	0.45	3.6
Bush and McInerney, 2015	47.23	-95.20	29.4	0.45	3.7
Bush and McInerney, 2015	47.23	-95.19	28.0	0.46	3.7
Bush and McInerney, 2015	47.54	-94.82	29.2	0.47	3.7
Bush and McInerney, 2015	48.12	-93.70	27.6	0.47	3.3
Bush and McInerney, 2015	46.32	-95.68	28.2	0.47	5.2
Bush and McInerney, 2015	45.54	-95.52	28.6	0.50	6.0
Bush and McInerney, 2015	44.73	-95.44	28.4	0.52	7.0
Bush and McInerney, 2015	45.25	-95.67	30.4	0.53	6.4
Bush and McInerney, 2015	43.89	-96.37	30.1	0.53	6.7
Bush and McInerney, 2015	43.85	-96.44	30.5	0.53	6.8
Bush and McInerney, 2015	44.35	-95.92	30.2	0.54	6.7
Bush and McInerney, 2015	43.69	-96.52	30.2	0.56	7.3
Bush and McInerney, 2015	43.22	-96.58	29.6	0.60	7.8
Bush and McInerney, 2015	42.92	-96.77	29.9	0.63	8.3
Bush and McInerney, 2015	41.86	-98.09	30.2	0.66	9.1
Bush and McInerney, 2015	42.90	-96.91	30.1	0.66	8.6
Bush and McInerney, 2015	42.15	-98.10	30.2	0.67	9.3
Bush and McInerney, 2015	42.15	-98.10	30.4	0.67	9.3
Bush and McInerney, 2015	42.51	-97.57	30.5	0.67	8.6
Bush and McInerney, 2015	41.45	-98.77	30.5	0.72	9.3
Bush and McInerney, 2015	41.27	-98.41	29.9	0.72	9.8
Bush and McInerney, 2015	40.93	-98.99	31.4	0.75	9.8
Bush and McInerney, 2015	40.33	-99.05	31.1	0.76	10.2
Bush and McInerney, 2015	40.65	-98.99	29.8	0.77	10.1
Bush and McInerney, 2015	40.00	-98.89	29.3	0.79	10.6
Bush and McInerney, 2015	39.94	-98.86	31.2	0.82	10.9
Bush and McInerney, 2015	39.59	-98.75	30.2	0.88	11.7
Bush and McInerney, 2015	39.24	-98.81	30.3	0.91	11.7
Bush and McInerney, 2015	38.68	-98.88	30.7	0.92	12.5
Bush and McInerney, 2015	38.30	-98.98	30.9	0.94	12.8
Bush and McInerney, 2015	37.73	-99.00	30.4	0.96	13.2
Bush and McInerney, 2015	37.65	-98.98	30.3	0.96	13.2
Bush and McInerney, 2015	37.63	-99.01	30.2	0.96	13.2
Bush and McInerney, 2015	37.46	-98.92	30.4	1.00	13.6
Bush and McInerney, 2015	25.85	-97.42	30.2	1.03	23.0
Bush and McInerney, 2015	25.86	-97.42	30.8	1.03	23.0
Bush and McInerney, 2015	36.22	-99.21	32.2	1.04	14.4
Bush and McInerney, 2015	35.96	-99.29	31.8	1.06	14.7
Bush and McInerney, 2015	36.53	-99.88	30.2	1.07	13.8
Bush and McInerney, 2015	26.00	-97.56	30.4	1.07	22.9
Bush and McInerney, 2015	35.75	-99.72	30.3	1.08	14.6
Bush and McInerney, 2015	35.75	-99.72	30.9	1.08	14.6
Bush and McInerney, 2015	30.02	-100.20	31.2	1.08	18.3
Bush and McInerney, 2015	36.70	-99.14	31.8	1.08	14.5

Table S1 contd: Modern compilation of soil data used to define the relationship between ACL and VPD

Data Source	Latitude	Longitude	ACL	VPD_{av} (kPa)	MAT (°C)
Bush and McInerney, 2015	36.83	-99.11	30.2	1.09	14.4
Bush and McInerney, 2015	35.31	-99.60	32.3	1.11	15.3
Bush and McInerney, 2015	34.87	-99.62	31.1	1.19	16.0
Bush and McInerney, 2015	34.44	-99.71	32.8	1.23	16.6
Bush and McInerney, 2015	29.48	-99.69	32.2	1.23	19.5
Bush and McInerney, 2015	29.63	-100.44	30.7	1.23	19.1
Bush and McInerney, 2015	32.27	-99.85	30.8	1.24	17.6
Bush and McInerney, 2015	32.23	-99.88	30.4	1.24	17.5
Bush and McInerney, 2015	32.23	-99.88	30.2	1.24	17.5
Bush and McInerney, 2015	28.48	-98.35	30.7	1.24	21.5
Bush and McInerney, 2015	30.44	-99.80	31.1	1.25	18.3
Bush and McInerney, 2015	30.82	-100.12	30.6	1.26	18.0
Bush and McInerney, 2015	27.23	-98.09	30.9	1.26	22.3
Bush and McInerney, 2015	32.04	-100.25	30.4	1.28	17.6
Bush and McInerney, 2015	27.78	-98.42	31.8	1.28	21.7
Bush and McInerney, 2015	31.47	-100.52	30.8	1.29	18.0
Bush and McInerney, 2015	32.98	-99.88	29.4	1.30	18.0
Bush and McInerney, 2015	33.74	-99.80	32.8	1.30	17.2
Bush and McInerney, 2015	34.10	-99.76	29.8	1.32	16.9
Bush and McInerney, 2015	31.50	-99.92	30.1	1.33	18.6
Bush and McInerney, 2015	29.02	-99.31	30.7	1.34	20.6
Douglas et al. 2012	14.12	-87.09	31.1	0.96	19.1
Douglas et al. 2012	14.35	-86.85	29.5	1.01	19.3
Douglas et al. 2012	15.74	-89.07	30.7	1.06	26.2
Douglas et al. 2012	14.89	-89.03	30.4	1.07	23.0
Douglas et al. 2012	14.94	-88.03	29.7	1.07	23.1
Douglas et al. 2012	15.1	-88.71	30.2	1.10	24.6
Douglas et al. 2012	14.71	-87.97	30.4	1.11	22.8
Douglas et al. 2012	15.41	-88.14	30.0	1.15	26.0
Douglas et al. 2012	14.84	-89.15	30.6	1.17	23.9
Douglas et al. 2012	14.26	-87.24	30.2	1.17	22.6
Douglas et al. 2012	14.48	-86.98	29.9	1.18	22.3
Douglas et al. 2012	14.45	-86.81	29.6	1.21	23.0
Douglas et al. 2012	20	-89.03	30.3	1.30	25.7
Douglas et al. 2012	15.12	-87.94	30.0	1.30	26.6
Douglas et al. 2012	20.16	-89.24	30.3	1.30	25.7
Douglas et al. 2012	14.45	-87.65	30.0	1.32	24.3
This study	39.54	-106.22	29.4	0.51	-0.1
This study	44.79	-107.94	28.9	0.51	2.7
This study	44.82	-112.00	29.4	0.52	1.5
This study	44.79	-107.39	28.9	0.56	2.2
This study	44.82	-113.25	28.9	0.56	1.8
This study	45.02	-112.25	29.5	0.58	3.7
This study	44.86	-112.50	29.2	0.59	2.3
This study	37.95	-107.84	29.6	0.67	2.6
This study	45.50	-111.70	29.9	0.70	5.7
This study	44.82	-107.34	29.9	0.70	4.9
This study	44.18	-112.22	29.3	0.74	5.9

Table S1 contd: Modern compilation of soil data used to define the relationship between ACL and VPD

Data Source	Latitude	Longitude	ACL	VPD_{av} (kPa)	MAT (°C)
This study	44.79	-107.98	30.0	0.76	4.7
This study	43.41	-112.22	28.9	0.77	7.0
This study	41.46	-104.88	30.4	0.78	7.6
This study	43.04	-112.21	29.9	0.79	6.3
This study	45.89	-111.61	28.9	0.79	7.2
This study	45.71	-110.40	30.1	0.80	7.5
This study	40.84	-104.96	30.1	0.81	8.7
This study	38.03	-108.11	30.7	0.82	6.8
This study	44.68	-106.87	29.9	0.84	7.1
This study	42.80	-112.26	30.6	0.85	7.0
This study	41.81	-104.80	30.1	0.86	8.3
This study	45.21	-108.87	29.9	0.87	8.0
This study	42.43	-105.04	30.3	0.88	8.2
This study	45.67	-109.25	29.9	0.89	7.7
This study	43.27	-106.39	30.1	0.90	7.3
This study	40.17	-104.99	29.8	0.94	9.7
This study	38.80	-104.79	29.1	0.95	9.6
This study	41.58	-112.23	29.7	0.96	9.8
This study	40.00	-111.41	30.3	0.98	7.8
This study	39.53	-107.71	29.0	0.99	8.7
This study	40.07	-111.62	29.7	1.06	9.9
This study	38.22	-108.49	31.2	1.11	10.0
This study	38.33	-109.43	30.3	1.18	10.6
Tipple and Pagani, 2013	-69.01	116	29.3	0.47	7.0
Tipple and Pagani, 2013	-68.65	56	28.7	0.49	6.7
Tipple and Pagani, 2013	-70.70	137	28.9	0.53	7.4
Tipple and Pagani, 2013	-71.78	209	28.4	0.54	9.0
Tipple and Pagani, 2013	-72.96	318	29.0	0.55	9.1
Tipple and Pagani, 2013	-73.50	156	29.2	0.55	9.7
Tipple and Pagani, 2013	-72.28	92	29.3	0.56	9.6
Tipple and Pagani, 2013	-71.16	95	29.3	0.57	8.5
Tipple and Pagani, 2013	-75.80	169	29.9	0.59	11.3
Tipple and Pagani, 2013	-74.44	82	29.9	0.64	11.2
Tipple and Pagani, 2013	-76.50	104	29.3	0.66	12.2
Tipple and Pagani, 2013	-76.76	179	29.5	0.66	12.2
Tipple and Pagani, 2013	-77.12	30	29.2	0.77	13.9
Tipple and Pagani, 2013	-77.12	33	29.2	0.78	14.7
Tipple and Pagani, 2013	-77.92	109	29.3	0.78	14.6
Tipple and Pagani, 2013	-77.59	89	28.7	0.78	14.5
Tipple and Pagani, 2013	-78.61	61	29.4	0.84	15.5
Tipple and Pagani, 2013	-78.37	112	29.5	0.85	14.9
Tipple and Pagani, 2013	-78.91	119	30.1	0.88	16.0
Tipple and Pagani, 2013	-80.49	67	29.8	0.92	18.0
Tipple and Pagani, 2013	-78.60	31	29.8	0.92	16.9
Tipple and Pagani, 2013	-81.09	37	30.0	0.93	18.4
Tipple and Pagani, 2013	-84.25	55	29.8	0.93	19.6
Tipple and Pagani, 2013	-78.60	28	30.0	0.93	17.0
Tipple and Pagani, 2013	-83.10	70	29.7	0.93	19.5
Tipple and Pagani, 2013	-82.13	60	29.6	0.95	19.1
Tipple and Pagani, 2013	-81.96	60	29.9	0.97	18.2

VPD is regulated, in part, by temperature and for a given hydrologic regime can show a clear linear relationship (Fig. S2). This is to be expected because the saturated vapor pressure of air (i.e. the maximum amount of water vapor the air can hold) is a function of temperature (Fig. S3) (Monteith and Unsworth, 1990). However, VPD is also affected by local or regional moisture dynamics, thus data from the distinct study areas included here have different MAT – VPD relationships (Fig. S2). Despite this, the presence of a genetic signaling pathway upregulating the production of leaf wax precursors in response to water deficit (Hooker et al., 2002), and the absence of a similar molecular response to increasing temperatures, strongly supports the conclusion that VPD, rather than MAT, exerts the dominant control on ACL values.

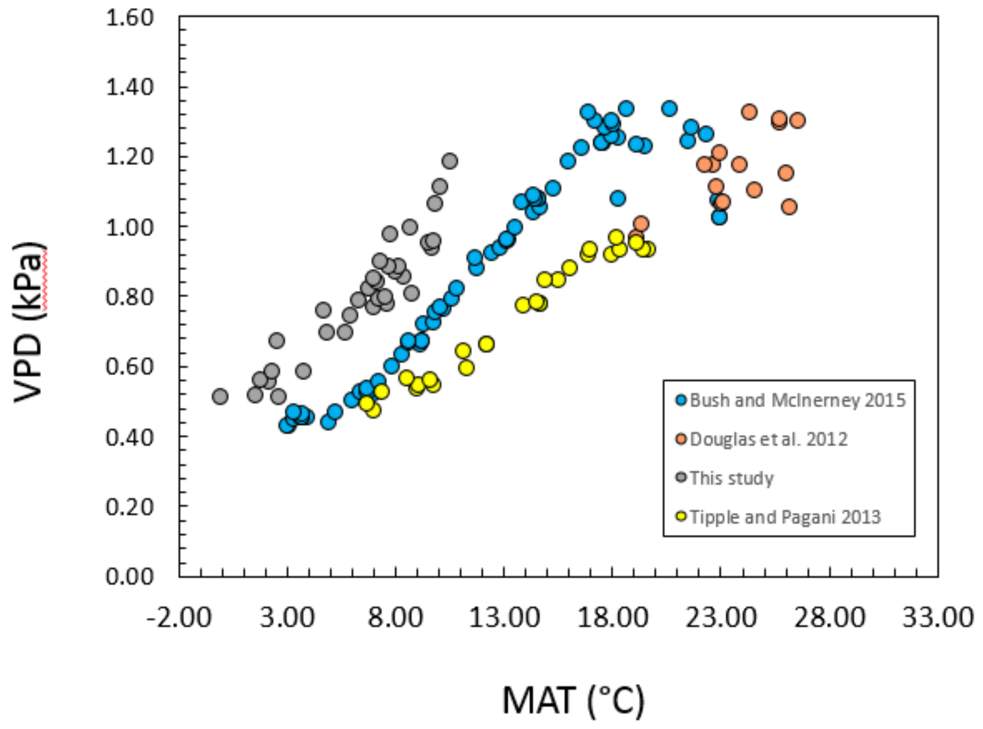

Figure S2: Relationship between mean annual temperature (MAT) and vapor pressure deficit (VPD) for the modern soil sampling locations.

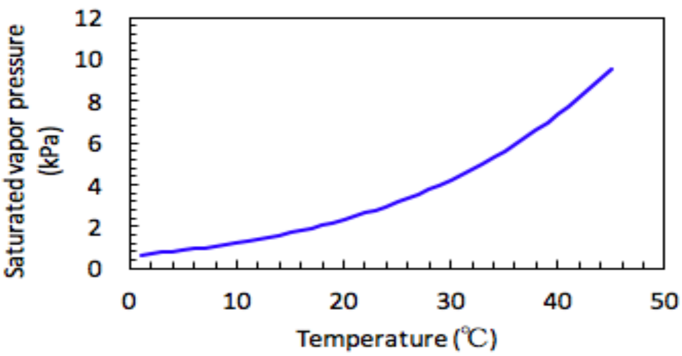

Figure S3: Relationship between temperature and the saturated vapor pressure of air (data from Monteith and Unsworth, 1990)

Regression analysis of ACL and VPD was carried out using Minitab v. 17. During early stages of model parameterization, a small number of samples with standardized residuals greater than 2.15 were identified as outliers, and removed from the final iteration of the model. There is a strong and statistically significant positive relationship (R^2 = 50.7%, p<0.05) between ACL and VPD_{av}. ACL values can therefore be used to predict annual average VPD values from the WRG samples using Eq. S2:

$$ACL = 26.78 + 5.670\ VPD - 2.162\ VPD^2 \qquad \textbf{Eq. S2}$$

The standard error of the regression (± 0.5 ACL units) translates to a 0.1kPa error on VPD reconstructions. This uncertainty was subsequently applied to all VPD reconstructions from the sediments from the Armantes Basin (Table S2).

Table S2: Organic molecular data from the Armantes section

Meter Level	ACL	Age (Ma)	VPD (kPa)
280	29.7	12.40	0.69
276	30.0	12.46	0.83
274	29.6	12.49	0.66
273	29.3	12.51	0.55
272	30.2	12.52	0.92
258	29.2	12.73	0.54
240	28.4	13.00	0.33
202	28.9	13.57	0.44
129	28.3	14.67	0.29
91	29.1	15.24	0.51
66	28.1	15.61	0.26
11	27.5	16.44	0.13

SUPPLEMENTARY REFERENCES:

Bush, R. T. & McInerney, F. A. 2015. Influence of temperature and C_4 abundance on n-alkane chain length distributions across the central USA. Org. Geochem*.* **79,** 65–73.

Douglas, P., Pagani, M., Brenner, M., Hodell, D., Curtis, J. 2012. Aridity and vegetation composition are important determinants of leaf-wax dD values in southeastern Mexico and Central America. Geochim. Cosmochim. Acta **97**, 24-45.

Fick, S. E. & Hijmans, R. J. WorldClim 2: new 1-km spatial resolution climate surfaces for global land areas. *Int. J. Climatol.* (2017).

Hooker, T.S., Millar, A.A., and Kunst, L., 2002, Significance of the expression of the CER6 condensing enzyme for cuticular wax production in Arabidopsis: Plant physiology, v. 129, no. 4, p. 1568–1580.

Monteith, J.L., Unsworth, M.H. (1990) *Principles of environmental physics.* Edward Arnold; London, etc.; 290 pp.

PRISM Climate Group (2010) Gridded climate data for the contiguous USA. http://prism.oregonstate.edu, accessed August 2016.

Tipple, B. J. & Pagani, M. 2013. Environmental control on eastern broadleaf forest species' leaf wax distributions and D/H ratios. Geochim. Cosmochim. Acta **111,** 64–77.
